# Sensitive detection and propagation of brain-derived tau assemblies in HEK293-based wild-type tau seeding assays

**DOI:** 10.1016/j.jbc.2025.108245

**Published:** 2025-01-27

**Authors:** Melissa Huang, William A. McEwan

**Affiliations:** UK Dementia Research Institute at the University of Cambridge, Department of Clinical Neurosciences, Cambridge, United Kingdom

**Keywords:** tau, tauopathy, seeded aggregation, Alzheimer's disease, biosensor assay

## Abstract

The assembly of tau into filaments defines tauopathies, a group of neurodegenerative diseases including Alzheimer’s disease (AD), Pick’s disease (PiD), corticobasal degeneration (CBD), and progressive supranuclear palsy (PSP). The seeded aggregation of tau has been modeled in cell culture using pro-aggregate modifications such as truncation of N- and C-termini and point mutations within the microtubule-binding repeat domain. This limits the applicability of research findings to sporadic disease, where aggregates contain wild-type, full-length tau. We describe a sensitive and specific biosensor assays for brain-derived tau species utilizing wild-type 0N3R and 0N4R tau expressed in HEK293 cells. We further generate a cell line that propagates AD-templated insoluble tau which is hyperphosphorylated at disease-relevant sites and retains a seeding profile similar to AD. We propose these systems as an advance over existing cell-based seeding assays, as they display specificity to the conformation and isoform composition of sporadic human disease.

Microtubule-associated protein tau assembles into highly ordered amyloid filaments in a subset of neurodegenerative diseases, termed tauopathies. Mutations in the autosomal dominant *MAPT* gene are causative of tau aggregation and subsequent neurodegeneration in frontotemporal dementia and parkinsonism linked to chromosome 17 (FTDP-17) (1, 2, 3). Studies utilizing these *MAPT* mutations have firmly established the importance of abnormal tau assembly to neuronal dysfunction and death. Mouse models transgenic for human P301S tau, a mutation causative of early-onset FTDP-17 (4, 5, 6, 7, 8, 9), develop hyperphosphorylated, filamentous tau inclusions and neurodegeneration (10, 11, 12). Importantly, the majority of neurodegenerative tauopathies develop independent of *MAPT* mutations. For example, accumulation of hyperphosphorylated tau aggregates occurs in Alzheimer’s disease (AD), sporadic Pick’s disease (PiD), corticobasal degeneration (CBD), and progressive supranuclear palsy (PSP) in the absence of *MAPT* mutations. The accumulation of tau aggregates in cases of “wild-type” tauopathies has nevertheless been shown to correlate with brain atrophy and the severity of dementia (13, 14).

Six isoforms of tau are expressed by alternative splicing of the *MAPT* gene in the central nervous system of healthy adults. Three isoforms exclude exon 10, which encodes the second repeat (R2) of the microtubule-binding repeat domain (3R tau), and three isoforms include all four repeat domains (4R tau) (15). Recent electron cryo-microscopy structures have shown that the core of the amyloid filaments found in tauopathies is comprised of the microtubule-binding repeat domains, while the N- and C-termini of tau protein remains largely unstructured (16). Consequently, the isoform composition of tau filaments can differ between different diseases. In AD, a mixture of 3R and 4R tau isoforms are present in the filaments, whereas 3R tau makes up the filaments found in PiD and 4R tau is found in CBD and PSP.

Studies of recombinant, purified tau protein have demonstrated that the wild-type full-length protein is highly soluble and requires the addition of negatively charged polyanionic co-factors (17, 18, 19) or agitation with glass beads (20) to form filaments *in vitro*. Tau filaments induced to form with heparin (21), phosphoserine, or RNA (22), have been shown to form structures distinct from those found in human disease. Recent advances have shown that, under specific assembly conditions, tau fragments consisting of microtubule-binding repeats can be induced to form paired helical filaments (PHFs) structurally identical to those found in AD (22). Full-length tau assemblies, however, make up the aggregates found in tauopathies but, to date, the spontaneous formation of full-length *in vitro* assembled PHFs has not yet been reported.

The seeded aggregation of full-length tau has been shown experimentally in various cell culture models, in which brain-derived or recombinant tau assemblies can enter the cellular environment to seed the subsequent aggregation of native tau (23, 24, 25). Such experiments have further shown that the conformation of the tau seeds dictates the potency of seeded aggregation (26), and that tau isolated from a 3R or 4R tauopathy specifically seeds mutant repeat domains of 3R or 4R tau, respectively (27). Further, mutant tau assemblies can preferentially promote aggregation of monomers bearing the same mutant over wild type (28). Despite this, the most commonly used “biosensor” assays for seeding competency of tau species rely on the P301S mutation on either truncated (29) or full-length (30) tau. The conformations resulting from seeding onto the P301S tau expressed in cell culture are unknown; however, individuals with P301L and P301T mutations, as well as transgenic mouse models expressing human P301S tau, develop tau filament structures distinct from those found in sporadic disease (31, 32). It is thus likely that the structure of the original seed is not accurately propagated by P301S mutant tau. On the other hand, Tarutani *et al*. demonstrated that full-length, wild-type 1N3R or 4R tau transiently expressed in undifferentiated SHSY5Y neuroblastoma cells could be seeded by brain-derived seed to propagate tau filament structures that maintain some structural properties of the original filaments found in AD and CBD.

We here develop a biosensor assay using HEK293 cells overexpressing full-length, wild-type 0N3R and/or 0N4R tau, which responds sensitively to brain-derived tau seeds and exhibits isoform-specificity for either 3R or 4R tauopathies. Further, we describe a cell line that retains hyperphosphorylated, AD-propagated insoluble tau over multiple passages. We demonstrate the use of these aggregate-containing HEK293 cells as a source of seed-competent tau assemblies that harbor PTMs similar to those found in disease. We show that our cell lines respond preferentially to AD brain-derived or AD HEK-propagated tau seeds over *in vitro* heparin-induced filaments, suggesting that the HEK-derived tau retains the biological properties of AD brain-derived tau.

## Results

**Figure 1:**
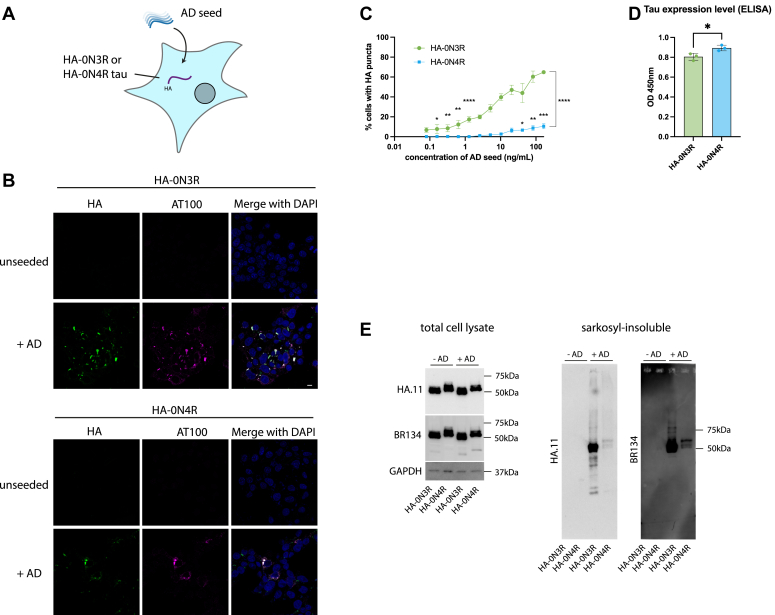
**Seeded aggregation of HA-tagged full-length, 0N3R, and 0N4R wild-type tau by AD brain-derived tau seed**. *A*, diagram depicting HEK293 cell line expressing HA-0N3R or HA-0N4R tau and seeded with AD seed. *B*, representative immunofluorescence images of HEK293 cells stably expressing HA-0N3R or HA-0N4R tau with or without treatment with AD seed in the presence of Lipofectamine 2000. sb = 10 μm. *C*, quantification of percent cells bearing HA-puncta (two-way ANOVA with Fisher’s LSD, ∗∗∗∗*p* < 0.0001, ∗∗∗*p* = 0.0002, ∗∗*p* < 0.006, ∗*p* < 0.03). *D*, total tau (HT7) ELISA showing relative expression levels of HA-0N3R and HA-0N4R HEK293 cell lines (unpaired *t* test, ∗*p* = 0.0278). *E*, immunoblot of total cell lysate and sarkosyl-insoluble fraction of HEK293 cell lines with or without treatment with AD seed using a C-terminal tau antibody (BR134) and anti-HA antibody.

We developed a HEK293 cell line stably expressing the 0N3R or 0N4R isoform of human tau with an N-terminal HA (YPYDVPDYA) tag by lentiviral transduction. Sarkosyl-insoluble tau assemblies extracted from AD patient brain (AD seed) were introduced to the cells using Lipofectamine 2000 (Fig. 1*A*). The addition of AD seed resulted in the formation of methanol-resistant HA-tau puncta which colocalize with an antibody selective for hyperphosphorylated, pathological tau (AT100), by immunofluorescence microscopy (Fig. 1*B*). We observed a seed-dependent increase in the percent of cells developing HA-tau puncta, with no puncta observed in the untreated control cells. Significant seeding of HA-tau puncta was detectable in the HA-0N3R expressing cell line at ±0.16 ng/ml of AD seed, which is equivalent to approximately 0.2 μg of brain, extracted, per well. We observed a significant difference in the seeding competency of HA-0N3R compared to HA-0N4R tau in response to AD seed at all concentrations where seeding was detected (*p* < 0.0001), consistent with previous reports in transiently transfected, undifferentiated SHSY5Y cells (33, 34) (Fig. 1*C*). The reason for this is not clear, although we speculate that the lack of the R2 domain in 3R tau could reduce this isoform’s interaction with microtubules, leaving it more prone to aggregation in comparison with 4R tau. This was despite the HA-0N4R expressing slightly more total tau than the HA-0N3R cell line (*p* = 0.0278) (Fig. 1*D*). We confirmed that the appearance of immunoreactive puncta corresponded to the accumulation of sarkosyl-insoluble HA-tagged tau upon the addition of seed (Fig. 1*E*). Thus, wild-type tau can be expressed in cell lines for the sensitive detection of seed-competent species from diseased human brain samples.

**Figure 2:**
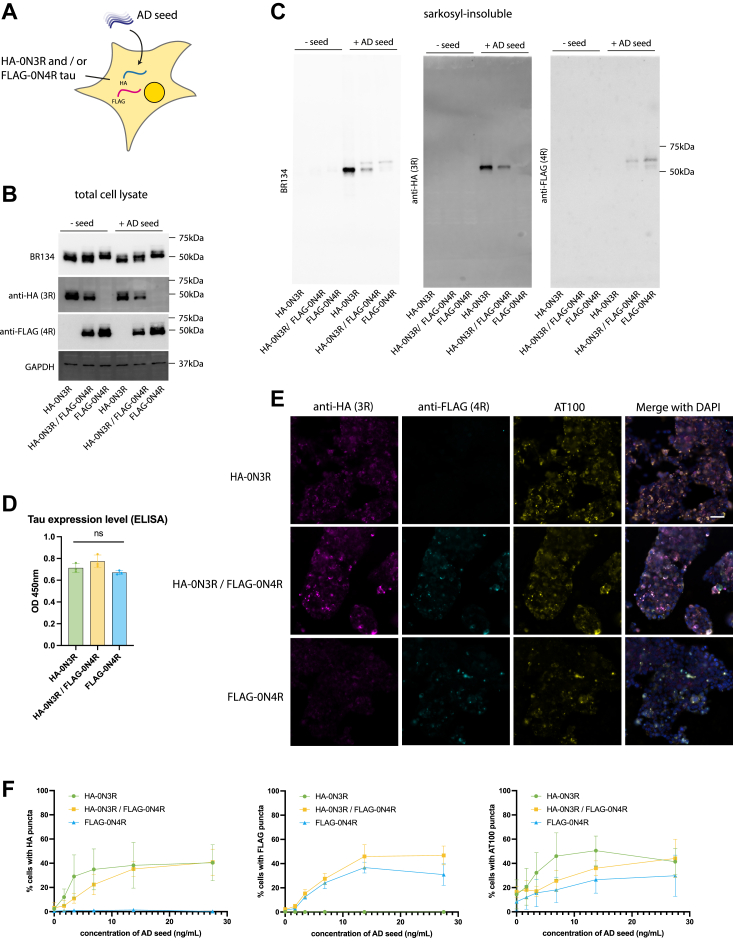
**Seeded aggregation of 3R and 4R tau in co-expressing cell lines.***A*, diagram depicting HEK293 cell line expressing HA-0N3R and/or FLAG-0N4R tau and seeded with AD seed. *B*, immunoblot images of total cell lysate of HEK293 cells expressing HA-0N3R, HA-0N3R/FLAG-0N4R, or FLAG-0N4R cells using C-terminal tau (BR134), anti-HA, and anti-FLAG antibodies. *C*, Immunoblot images of sarkosyl-insoluble fraction of HEK293 cells expressing HA-0N3R, HA-0N3R/FLAG-0N4R, or FLAG-0N4R cells using C-terminal tau (BR134), anti-HA, and anti-FLAG antibodies and seeded with AD. *D*, total tau (HT7) ELISA showing relative expression levels of HA-0N3R, HA-0N3R/FLAG-0N4R, or FLAG-0N4R cell lines. Ordinary one-way ANOVA (*p* > 0.05). *E*, representative immunofluorescence images of cell lines seeded with AD using AT100 (pS212/pT215), anti-HA, and anti-FLAG antibodies. *F*, quantification of the percentage of seeded cells with a titration of AD seed using anti-HA, anti-FLAG, or AT100 antibodies.

In the adult brain, 3R and 4R tau isoforms are expressed at similar levels (15), and in AD patients, tau inclusions contain both 3R and 4R tau. To test whether co-expression of 3R and 4R tau altered the response to exogenous seeds, we generated a cell line expressing both HA-0N3R tau and FLAG-0N4R tau (HA-0N3R/FLAG-0N4R) and compared it to cells expressing either construct alone (Fig. 2*A*). Total tau expression in HA-0N3R, FLAG-0N4R, and HA-0N3R/FLAG-0N4R was equal (Fig. 2, *B* and *D*), with the decreased individual expression of HA-0N3R and FLAG-0N4R in the co-expressing line (Fig. 2*B*). The addition of AD seed resulted in the formation of sarkosyl-insoluble tau (Fig. 2*C*) in HA-0N3R, FLAG-0N4R, and HA-0N3R/FLAG-0N4R cells with two distinct bands in the co-expressing cell line corresponding with the molecular weights of 0N3R and 0N4R tau. Immunocytochemistry using AT100 and antibodies specific to HA or FLAG showed an AD seed concentration-dependent formation of AT100+/HA+ methanol-resistant puncta in the HA-0N3R and HA-0N3R/FLAG-0N4R cell lines, and AT100+/FLAG+ puncta in the FLAG-0N4R and HA-0N3R/FLAG-0N4R cell lines (Fig. 2, *E* and *F*). HA and FLAG puncta largely co-localized in the co-expressing cell line (Fig. 2*E*). No significant differences were observed in the percentage of seeded cells across the three cell lines (Fig. 2*F*). Thus, co-expression of 3R and 4R isoforms does not further increase efficiency of seeded aggregation beyond that observed with single isoform expression.

**Figure 3:**
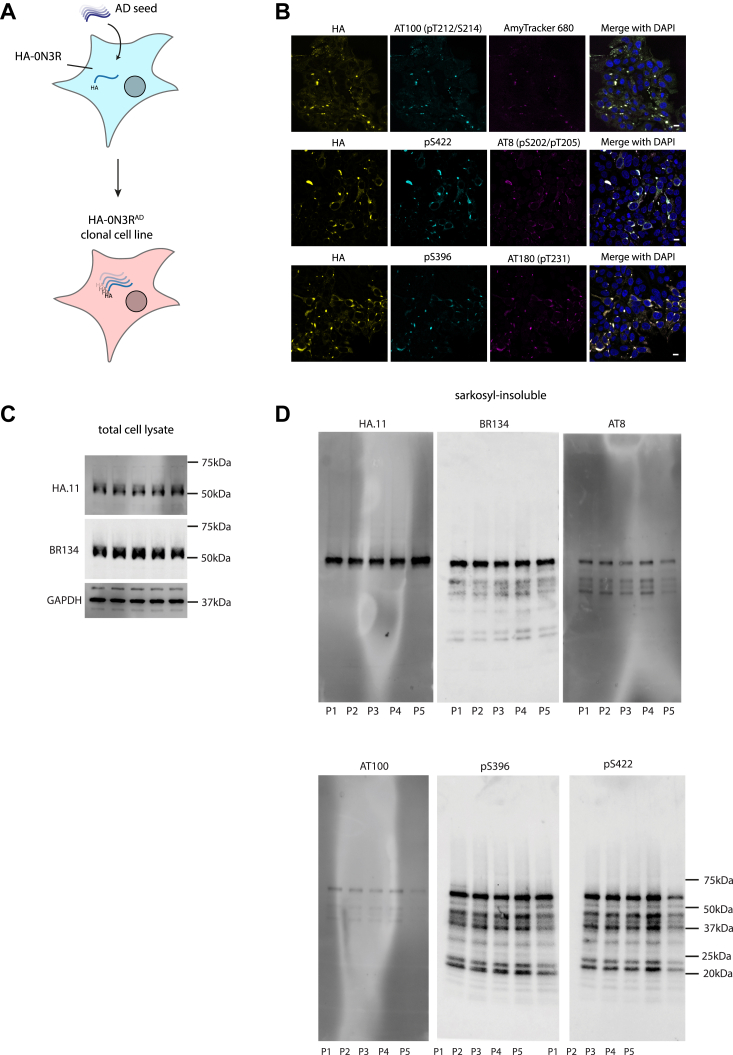
**HA-0N3R**^**AD**^**HEK293 cells propagate AD-brain-derived tau seeds**. *A*, diagram depicting HEK293 cell line expressing HA-0N3R, which has been clonally selected to retain AD-seeded aggregates (HA-0N3R^AD^). *B*, representative immunofluorescent images of HA-0N3R^AD^ cells at passage 4 (P4), probing with phosphorylation-specific antibodies: AT180, AT8, AT100, pS396, and pS422, and with an amyloid-specific dye, Amytracker 680. sb = 10 μm. *C*, immunoblot of total cell lysate HA-0N3R^AD^ cells over multiple passages. *D*, immunoblot of the HA-0N3R^AD^ cells over multiple passages using a C-terminal tau antibody (BR134), anti-HA antibody, and phosphorylation-specific antibodies: AT8. AT100, pS396, pS422. Sarkosyl-insoluble extract from approximately 300,000 cells was loaded into each lane.

**Figure 4:**
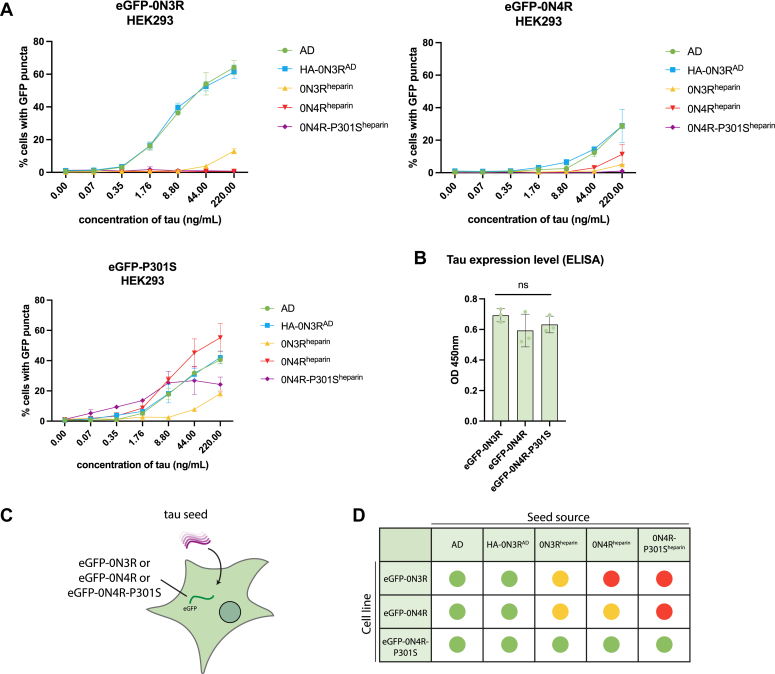
**WT-tau expressing cell lines respond preferentially to AD-brain derived seed.***A*, levels of seeded aggregation in HEK293 cells expressing the indicated eGFP-tagged tau construct following treatment with AD-brain derived tau assemblies, with AD-HEK derived tau (HA-0N3R^AD^) or with recombinant heparin tau assemblies (0N3R^heparin^, 0N4R^heparin^, 0N4R-P301S^heparin^). *B*, total tau ELISA showing relative expression levels of eGFP-0N3R, eGFP-0N4R, and eGFP-0N4R-P301S HEK293 cells (One-way ANOVA with Tukey’s multiple comparisons test, *p* > 0.05). *C*, a diagram depicting HEK293 cell line expressing eGFP-0N3R, 0N4R, or 0N4R-P301S and seeded with various sources of tau seeds. *D*, a graphical depiction of the seeding competency of tau species on expressing eGFP-0N3R, 0N4R, or 0N4R-P301S cell lines. *Green* indicates robust seeding, *yellow* indicates low levels of seeding at high concentrations, and *red* indicates no detectable seeding.

We next asked whether the aggregated state following seeding with AD tau could be inherited during cell division to derive a replenishable source of tau assemblies. Clonal populations of cells were isolated from HA-0N3R-expressing cells seeded with AD brain by limiting dilution. Clones were screened by immunofluorescence microscopy for persistent HA-tagged puncta, and a positive clone was selected and expanded (HA-0N3R^AD^) (Fig. 3*A*). HA-0N3R^AD^ HEK293 cells contain HA-tagged tau aggregates which are hyperphosphorylated at pT231 (AT180), pS202/pT205 (AT8), pT212/pS214 (AT100), pS396, pS422, and are positive for AmyTracker 680, an amyloid-specific dye (Fig. 3*B*). Additional treatment with AD seed, followed by cell expansion resulted in cells that maintain insoluble, hyperphosphorylated tau aggregates over multiple passages (Fig. 3, *C* and *D*). To investigate the utility of these cells as a source of hyperphosphorylated tau seeds, we incubated eGFP-0N3R or 0N4R expressing HEK293 cells with sarkosyl-insoluble tau extracted from AD brain or HA-0N3R^AD^ HEK293 cells (HA-0N3R^AD^ seed) (Fig. 4*C*). The use of a distinct tag (eGFP) ensures that the original HA-0N3R^AD^ seed is not the source of the signal. In both cell lines, AD seed, and HA-0N3R^AD^ seed behaved similarly, with equal amounts of assembled tau inducing similar percentages of seeded cells, as measured by eGFP puncta, at all concentrations (*p* > 0.05) (Fig. 4*A*). Heparin-assembled tau filaments are frequently used a source of tau seeds in place of brain-derived material to study the seeded aggregation of tau. While heparin-assembled recombinant 0N3R, 0N4R, and 0N4R-P301S tau (0N3R^heparin^, 0N4R^heparin^, 0N4R-P301S^heparin^) seeded aggregation in cells expressing eGFP-0N4R-P301S tau, we observed that only 0N3R^heparin^ was able to seed aggregation in eGFP-0N3R cells. Both 0N3R^heparin^ and 0N4R^heparin^ tau assemblies were able to seed aggregation in eGFP-0N4R cells, albeit requiring higher concentrations than for AD seed or HA-0N3R^AD^ seed. 0N4R-P301S^heparin^ assemblies were incompatible with seeding in both eGFP-0N3R and 0N4R cells. HA-0N3R^AD^ seed exhibited a greater level of seeding than heparin-assembled tau at all concentrations where seeding was detected (Fig. 4*A*). All three cell lines expressed similar levels of total tau (Fig. 4*B*). Thus, cells expressing P301S tau are responsive to all sources of seed tested. In contrast, cells expressing wild-type tau supported seeding in response to brain-derived seeds but exhibited poor seeding in response to heparin assemblies (Fig. 4*D*). AD seed or HEK293 propagated AD-seeded tau assemblies were similarly active in wild-type tau-expressing cells, suggesting shared biological properties. We observed similar results with AD and 0N4R-P301S^heparin^ seed when comparing the eGFP-0N3R cell line to the commonly used biosensor cell line expressing the repeat domains of tau with the P301S mutation (29). Neither cell line was seeded following the addition of recombinant tau monomer (Fig. S1).

**Figure 5:**
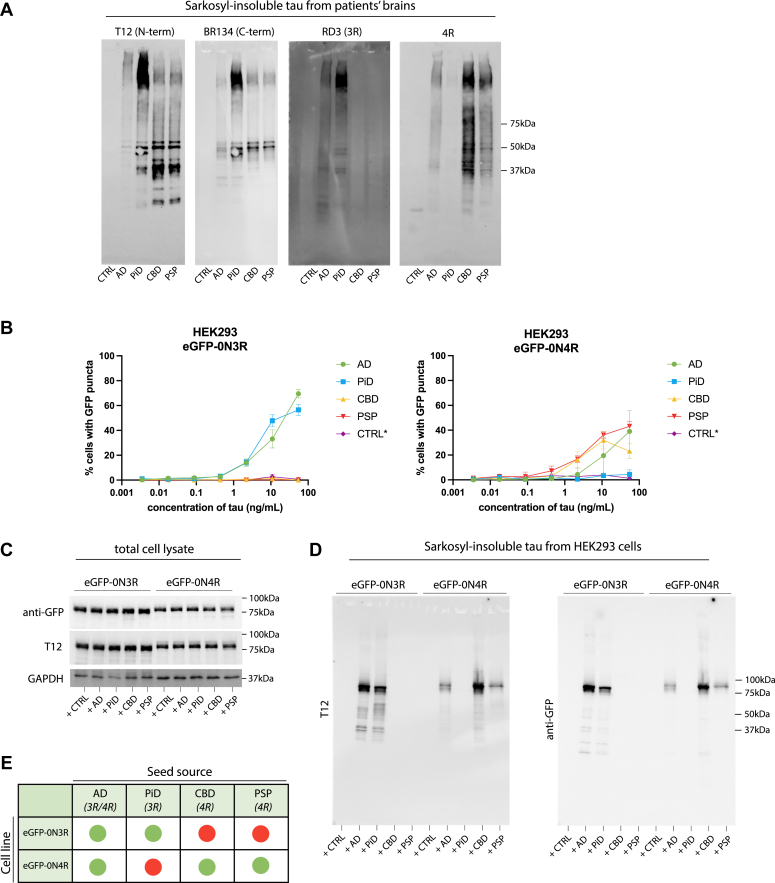
**Wild-type tau-expressing cell lines exhibit isoform specificity for 3R or 4R tauopathies**. *A*, immunoblotting of the sarkosyl insoluble fraction from control, AD, PiD, CBD, and PSP brain tissue using antibodies against the N-terminus (T12) and C-terminus (BR134) of tau, 3R tau (RD3) and 4R tau (anti-4R). *B*, HEK293 cells engineered to express either 0N3R or 0N4R tau with an N-terminal eGFP tag were treated with sarkosyl-insoluble fractions prepared from control (CTRL), AD, PiD, CBD, and PSP brains in the presence of Lipofectamine 2000. Concentration of brain-origin insoluble tau was normalized following quantification using the total tau ELISA kit (ab273617). ∗ For the control brain extract, the mean volume of seed for the disease brain extracts was used, as no sarkosyl-insoluble tau was detected by ELISA. *C*, immunoblot analysis of total cell lysates and (*D*) sarkosyl-insoluble fractions of eGFP-0N3R and eGFP-0N4R HEK293 cells treated with control, AD, PiD, CBD, and PSP brain extract using antibodies against the N-terminus (T12) of tau and anti-GFP. *E*, graphical depiction of the seeding competency of AD, PiD, CBD, and PSP on HEK293 cells expressing eGFP-0N3R or 0N4R tau.

While the AD filament fold excludes the R1 and R2 domain of the microtubule-binding repeat domains, and can thus incorporate both 3R and 4R isoforms in its structure, other sporadic tauopathies have filament structures that allow incorporation of only 3R (PiD) or 4R (CBD, PSP) tau isoforms (35). Isoform-specific seeded tau aggregation of full-length, wild-type tau has been shown previously in transiently transfected SH-SY5Y cells (33). We tested whether our HEK293 stable cell lines also respond with isoform specificity to 3R or 4R tau strains. We extracted sarkosyl-insoluble tau from AD, PiD, CBD, and PSP patient brain tissue, and confirmed the presence of 3R or 4R insoluble tau by immunoblotting (Fig. 5*A*). Addition of sarkosyl-insoluble tau extracted from PiD patient brain specifically seeded the eGFP-0N3R cell line (Fig. 5*B*) and tau extracted from CBD or PSP patient brain specifically seeded the eGFP-0N4R cell line (Fig. 5*B*). We confirmed that the cells develop sarkosyl-insoluble GFP-tagged tau upon the addition of seed (Fig. 5*C*). These results demonstrate that reporter cell lines expressing wild-type tau exhibit isoform-specific aggregation (Fig. 5*E*).

## Discussion

In addition to the eight known wild-type tau structures associated with human disease (16) around fifty conformations of tau, or its truncation products, have been reported to arise during *in vitro* aggregation (21, 22, 36). The biological significance of these structures remains unclear, but the observation that particular folds are associated with specific diseases raises the possibility that tau filament conformation is closely tied to its pathogenesis. Given that mutations in the amyloid core region of tau are likely to have a consequential effect on the structure of tau filaments (31), methods that enable the propagation of wild-type tau are required to effectively model brain-derived tau species. Here we have developed a biosensor assay in the widely used HEK293 cell line, where wild-type tau responds to exogenously supplied tau assemblies extracted from post-mortem patient brain tissue. Aggregation is highly sensitive, with tau isolated from 0.2 μg of AD brain tissue sufficient to yield significant seeding. This finding is surprising, given the widely held view that tau mutants or truncations are required to observe seeded aggregation, particularly at low concentrations of supplied tau seeds. In contrast to cell lines expressing mutant tau, our wild-type tau-expressing reporter lines respond preferentially to brain-derived tau assemblies over heparin-assembled recombinant tau. This suggests that our wild-type cell lines have greater specificity for disease-relevant conformations, while P301S cell lines are less specific and thus less likely to retain structural information from the initial seed.

Full-length tau is highly soluble, and the assembly of recombinant tau into seed-competent filaments for experimental procedures is most commonly achieved using anionic cofactors such as heparin or arachidonic acid (18, 37, 38). The truncated core of the AD protofilament (amino acids 297–391) has been shown to form paired helical filaments (PHFs) identical to those found in AD brain (22, 39), but, to date, full-length recombinant tau has not been reported to form disease-relevant structures. A possible explanation for this is that recombinant tau assemblies suffer from a lack of post-translational modifications, which are highly enriched in patient brains (40) and likely play an important role in tau aggregation. Generating a brain-independent source of post-translationally modified tau seeds is thus an important step to modeling and understanding tau aggregation. Unlike *Escherichia coli*, HEK293 cells can produce protein with extensive post-translational modification. Previous work has demonstrated that aggregated tau may be inherited from mother-to-daughter cells in HEK293 cells expressing tau residues 244 to 372 (41). We here extend these findings by demonstrating that wild-type HA-0N3R tau can propagate hyperphosphorylated, insoluble tau assemblies during subsequent rounds of cell division following the addition of brain-derived tau assemblies. Importantly, the extracted tau from these cells behaves near-identically to AD brain-derived tau in its ability to further induce seeded aggregation.

This finding is contrary to a recent report that AD-propagated tau species using recombinant protein are not seed-competent (42). It is possible that tau propagated under cell-free *in vitro* conditions does not possess the qualities required for seeded aggregation to occur, such as post-translational modifications. Aggregates derived from HA-0N3R^AD^ cells, on the other hand, are both seed-competent and retain a phosphorylation status consistent with insoluble tau found in the brain of patient with AD(40). Our study is the first demonstration that full-length, wild-type tau can be propagated in this way. Extended cell passaging of these cell lines past P5 may result in a reduction in insoluble tau, as aggregates could be lost during cell division. We suspect that, over time, cells without aggregates may preferentially survive and proliferate in culture. While our findings are consistent with the structural properties of AD tau being retained, one limitation is that their structure is currently unknown. Tarutani *et al*. showed that undifferentiated SH-SY5Y cells, transiently transfected to express full-length 1N3R and 1N4R, and seeded with AD formed a single protofilament of the AD fold (33). We hypothesize that the structure propagated in HEK293 cells will be similar, however further structural studies should be performed.

In conclusion, we demonstrate that commonly used tau mutations are not required to develop sensitive seeding assays when seeds derived from the human brain are used. The wild-type seeding assays we developed display both isoform and conformer specificity. For isoform specificity, the number of repeats must be matched between seed and substrate in order to elicit robust seeding. For instance, PiD seeds did not induce aggregation in 4R tau-expressing cells whereas PSP and CBD did induce aggregation of 3R tau. AD tau was able to induce aggregation in cells expressing either 3R or 4R or both 3R and 4R. In terms of conformational specificity, we found that cells expressing wild-type tau display a strong preference to brain-origin seeds over heparin assemblies. These observations are compatible with a model where the successful propagation of tau assemblies is dependent on the fold of the tau seed in cells expressing wild-type tau. In contrast, cells expressing mutant tau could be induced to aggregate by both brain and laboratory tau preparations and did not display isoform specificity. We therefore propose that mutant tau cell lines offer convenience, as they have the potential to detect diverse tau conformers and do not require isoform matching. However, they do not capture the strain-type specificity that is present among human diseases. We propose wild-type tau seeding assays are necessary where such information is important. We recently showed that this system of overexpressing full-length, wild-type tau in HEK293 cells to propagate brain-derived species can be successfully utilized to show isoform-specific degradation of AD and PSP tau (43). We further demonstrated that continued propagation of AD-seeded cells permits extraction of tau assemblies with seeding and phosphorylation characteristics similar to brain-origin AD tau. As a replenishable resource, HA-0N3R^AD^ cells therefore represent an alternative to the extraction of tau assemblies from the post-mortem brain, which may facilitate research on tau aggregation.

## Method

### Cloning

Cloning was performed using In-Fusion (Takara) or restriction cloning. Constructs encoding 0N3R or 0N4R tau were tagged at the N-terminus with HA or eGFP tag. The pSMPP backbone (Addgene #104970) was modified by replacing the puromycin resistance gene with a neomycin resistance gene (pSMPN). Tagged-tau was subcloned into the lentiviral backbone by digestion at Mlu1/Not1 restriction sites (Fig. S2).

### Human embryonic kidney cell maintenance

HEK293 and HEK293T cell lines were maintained in Gibco DMEM + Glutamax + 10% FBS + 1% penicillin-streptomycin in a tissue culture incubator at 37°C with 5% CO_2_.

### Lentivirus production

Lentivirus was produced in HEK293 T cells mediated by Lipofectamine 3000 (Invitrogen, L3000001) according to the manufacturer’s instructions for lentivirus production using pMD2.g envelope (Addgene #12259), pCRV-gag-pol packaging plasmid (a gift from Prof Stuart Neil, Kings College London) and pSMPP or pSMPN transfer plasmid backbone. HEK293 cells were transduced for 72 h mediated by polybrene (Merck, TR10003) before selection using 1ug/ml puromycin dihydrochloride (Gibco, A1113803) or 0.25 mg/ml geneticin (Gibco, 10131027). Cell lines were made clonal by limiting dilution. For the co-expressing cell line, HEK293 cells were first transduced and selected with puromycin-resistant HA-0N3R lentivirus, before transduction and selection of neomycin-resistant FLAG-0N4R lentivirus.

### Preparation of sarkosyl-insoluble tau from human brain samples

*Postmortem* brain tissue and patients were obtained from the Cambridge Brain Bank under the ethically approved protocol: REC 16/WA/0240 (PSP and CBD) and NRES/20/EE/0183 (PiD). The donors were a 37-year-old male dying of renal failure secondary to type 1 diabetes, with a neuropathological diagnosis of “normal”, a 74-year-old female with confirmed neurobiological diagnosis of CBD, and an 85-year-old male with a confirmed neuropathological diagnosis of PSP. *Postmortem* brain from a patient with AD was obtained from the Oxford Brain Bank (Ethics approval reference: 15/SC/0639), UK Sound Central Oxford C Research Ethics Committee. The donor was an 81-year-old female with AD neuropathological diagnosis. Frozen brain tissue from frontal cortex (1.2–2.4 g) was homogenized in 10X (v/w) of extraction buffer containing 800 mM NaCl, 10 mM Tris-HCl pH 7.4, 2.5 mM EDTA, 10% sucrose, 2% sarkosyl + Pierce Protease and Phosphatase Inhibitor Mini Tablets (Thermo Scientific, A32959), using T 10 basic ULTRA-TURRAX, (IKA 9993737002). Tissue homogenate was incubated at RT for 1 h on a roller, then centrifuged at 13,000g for 20 min at RT (room-temperature). The supernatant was filtered through a 0.45 μm cell strainer and transferred to 1.5 ml ultracentrifuge tubes, then spun at 124,500g for 1 h at RT in a TLA-55 rotor and Beckman Coulter Optima MAX-XP ultracentrifuge. Pellets were combined and resuspended in 1 ml extraction buffer and re-centrifuged at 124,500g for 1 h. Pellet(s) was [combined and] resuspended in 1 ml 50 mM Tris-HCl, 150 mM NaCl. The resuspended pellet was re-ultracentrifuged as before, and the resulting pellet was resuspended in 250uL/g starting weight and sonicated in a water-bath sonicator for 20 cycles of 15 s on/5 s off. The concentration of sarkosyl-insoluble tau was determined by the Human Tau ELISA Kit (ab273617).

### Preparation of sarkosyl-insoluble tau from HEK293

Media was removed and cells were washed 2x with PBS. Cells were lysed in 250 μl/well of a 6-well plate of cell extraction buffer (800 mM NaCl, 10 mM Tris-HCl pH 7.4, 2.5 mM EDTA, 15% sucrose, 1% sarkosyl + Protease and Phosphatase Inhibitor (Pierce)) and collected by repeated pipetting. Cells were further lysed by sonication with a metal probe Q55 sonicator (Q55–110) using a one-eighth” diameter probe (#4422) (10% amplitude, 15 s), before incubating at 37°C for 30 min with shaking. Lysate was spun at 124,500g for 1 h at RT in a TLA-55 rotor. The pellet was washed 2x in TBS before resuspending in the final volume (6uL of TBS per well of a 6-well plate). The amount of sarkosyl-insoluble tau was estimated by the Human Tau ELISA Kit (ab273617).

### Production of heparin-assembled tau filaments

Human 6xHis-tagged 0N3R, 0N4R, and 0N4R-P301S tau in a bacterial expression vector (pRK172) was transformed in BL21 (DE3) *E*. *coli*, and colonies were used to inoculate 4 L of 2XTY media. Bacterial cultures were grown at 37°C, 200 RPM to OD600 = 0.5, followed by induction with 1 mM IPTG and incubation overnight at 16°C, 200 RPM. Cells were harvested by centrifugation at 5000*g* for 10 min at 4°C and resuspended in 10 ml/g of pellet with lysis buffer (25 mM HEPES pH7.4, 300 mM NaCl, 20 mM Imidazole, 1 mM Benzmidine, 1 mM PMSF, 14 mM *β*-mercaptoethanol, 1% NP-40 with Pierce protease and phosphatase inhibitor tablets (Thermo Scientific, A32959). Cell lysis was performed by boiling for 15 min, followed by clearing of the lysate by ultracentrifugation at 100,000g for 50 min at 4°C. Cleared lysate was loaded onto a His/TRAP HP column (GE Healthcare) and eluted with a linear gradient of imidazole. Fractions were analyzed by SDS-PAGE and tau-containing fractions were pooled and purified with a Nickel affinity column followed by size-exclusion chromatography. Tau protein was loaded onto a Cytive HiLoad 16/600 Superdex 200pg column (11,397,490), prequillibrated with TBS (150 mM NaCl, 50 mM Tris HCl) + 1 mM DTT.

Heparin-induced aggregation of tau was carried out according to previously published methods (21). Briefly, 60 *μ*M of recombinant tau in aggregation buffer (25 mM HEPES pH 7.4, 10 mM KCl, 5 mM MgCl_2_, 3 mM TCEP) was incubated with 20 *μ*M heparin at 37°C with 700 RPM shaking for 72 h. An aliquot of aggregation mixture was used to monitor aggregation with 15 *μ*M of ThioflavinT by fluorescence in a Varioskan LUX Multimode Microplate Reader (Thermo Fisher Scientific). The final aggregation mixture was ultracentrifuged at 55,000 RPM for 1 h to pellet tau assemblies, and the supernatant was discarded. The amount of heparin-assembled tau assemblies was estimated by the Human Tau ELISA Kit (ab273617) for an approximate comparison to brain-derived sarkosyl-insoluble material.

### Immunoblotting

Cell lysate or sarkosyl-insoluble tau was boiled for 5 min at 95°C in 1X NuPage LDS Sample Buffer (ThermoFisher) + 4% BME. Samples were run on 4 to 20% Novex Tris-Glycine gels (ThermoFisher Scientific) for 50 min at 200 V and transferred onto PVDF membranes using the Trans-Blot Turbo Transfer system (BioRad). Membranes were blocked in TBS + 0.1% Tween-20 + 5% milk for 1 h and incubated overnight at 4°C in primary antibody or for 2 h at RT. Following washes in TBS + 0.1% Tween-20, blot was incubated in anti-mouse StarbrightBlue 700 (BioRad #12004158, 1:2500), anti-rabbit DyLight 800-conjugated secondary antibody (Cell Signaling Technology, 5151, 1:10,0000), and/or anti-goat IgG HRP (ROCKLAND, eB270, 1:1000) for 45 min. The signal was visualized using near IR fluorescence detection or chemiluminescence (ChemiDoc MP, BioRad). The following primary antibodies were used for immunoblotting: Tau 12 (Sigma-Aldrich, MAB2241, 1:2000); BR134 (in-house, rabbit polyclonal, 1:4000); RD3 (Sigma-Aldrich, 05–803, 1:1000); Tau 4R (Cell Signaling Technology; 30,328); anti-GFP (Proteintech, 50430-2-AP, 1:2000); anti-HA.11 (Biolegend, 901516l; 1:2000); anti-FLAG (Sigma-Aldrich, F7425, 1:2000); anti-GAPDH (ThermoScientific, MA5-15738, 1:2000 or Sigma-Aldrich, MAB374, 1:5000); anti-CypA (Bio-techne; AF3589;1:1000); AT100 (pT212/pS214) (Invitrogen, MN1060, 1:500); AT8 (pS202/T205) (Invitrogen, MN1020, 1:500); AT180 (pT231) (Invitrogen, MN0140, 1:500); anti-pS396 (Novus Biologicals, NB100–82243, 1:500); anti-pS422 (Sigma, SAB4300215, 1:500).

### Full-length, wild-type tau HEK293 seeding assay

96 Well Black Polystyrene plates (Corning, CLS3603) were coated with Poly-D-Lysine (Gibco, A3890401) for 30 min at 37°C, before washing the plate 3x with 100 μl/well of PBS. Tau assemblies were incubated at 2X final concentration in OptiMEM Reduced Serum Media (Gibco, 31,985,062) in a 50 μl/well reaction mix with a 1:50 dilution of Lipofectamine 2000 (Invitrogen, 11,668,019) for 20 min at RT. Cells were trypsinized and counted using Countess II Automated Cell Counter (Invitrogen). Cells were diluted to 40,000 cells/ml in OptiMEM, and 50uL of cells was combined with 50uL of tau assembly mixture per well for a final concentration of 1:100 Lipofectamine 2000, 1X tau, and 20,000 cells/well. Cells were returned to the incubator and, after 1 h, 100 μl/well of DMEM + 10% FBS was added to each well. Seeding was allowed to occur for 72 h before analysis.

### Immunocytochemistry

HEK293 cells were fixed and permeabilized in 100% ice-cold methanol. Cells were blocked with PBS + 2% BSA and incubated in the primary antibody at RT for 2 h, or 4°C overnight. Cells were washed 3x in PBS and incubated in secondary antibody or streptavidin: Alexa Fluor 568 goat anti-mouse IgG (H  + L) Highly Cross-Adsorbed (Invitrogen, A-11031, 1:1000); Streptavidin Alexa Fluor 488 (Invitrogen, S11223, 1:1000); Alexa Fluor 647 goat anti-rabbit IgG (H  + L) Highly Cross-Adsorbed (Invitrogen, A-21245).

The following primary antibodies were used for immunocytochemistry FITC-anti-HA (Roche, 11,988,506,001, 1:200, discontinued); biotin-anti-HA (Roche, 12,158,167,001, 1:500); anti-FLAG (Sigma-Aldrich, F7425, 1:500), AT100 (pT212/pS214) (Invitrogen, MN1060, 1:500); AT8 (pS202/T205) (Invitrogen, MN1020, 1:500); AT180 (pT231) (Invitrogen, MN0140, 1:500); anti-pS422 (abcam, ab79415, 1:1000); anti-pS396 (Fisher Scientific, 44–752-G, 1:1000).

Nuclei were stained with PUREBLU Hoechst 33,342 (BioRad, 1,351,304). Amyloid filaments were labelled with Amytracker 680 (Ebba Biotech, 1:500).

### Indirect ELISA

For total tau expression analysis, cell lysate was immobilized on 96-well High-binding ELISA plates (Corning, 3590) in 1X ELISA Coating Buffer overnight at 4°C. The plate was washed 4x with PBS + 0.05% Tween-20 and blocked for 2 h at RT in 5% milk in PBS. The blocking buffer was removed and HT7 primary antibody (MN1000) was diluted in 5% milk/PBS to a final concentration of 0.1 μg/ml. The primary antibody was incubated on the plate for 1 h at RT, then washed 4x with PBS + 0.05% Tween-20. The plate was incubated with HRP- goat anti-mouse IgG (H  + L) (Invitrogen, A28177, 1:1000) in 5% milk for 1 h at RT, then washed 4x with PBS + 0.05% Tween-20. TMB substrate (Cell Signaling Technology, 7004) was added for 30 min for signal detection and quenched with 0.16 M H_2_SO_4_ before reading at 450 nM on a Varioskan LUX microplate reader (Thermo Scientific).

### Microscopy

Confocal images were acquired on an SP8 Lightning Confocal Microscope (Leica) with a 63x lens. Images for % seeding analysis were acquired on a Ti2 High Content Microscope (Nikon) with a 10x lens and analyzed using NISElements software.

### Statistical analysis

Statistical analysis was performed using GraphPad Prism software. All the data are shown as the mean ± SD.

## Data availability

All data are available within the manuscript.

## Supporting information

This article contains supporting information.

## Conflict of interest

The authors declare the following financial interests/personal relationships which may be considered as potential competing interests:

NMH was funded by Aprinoia Therapeutics. WM is a founder and consultant to TRIMTECH Therapeutics. WM has received funding from Takeda Pharmaceuticals for work unrelated to this study.
