# Supplementary material for: Sensitive detection and propagation of brain-derived tau assemblies in HEK293-based wild-type tau seeding assays[image]
Source: J Biol Chem. 2025 Jan 27;301(3):108245. doi: 10.1016/j.jbc.2025.108245 (PMC11910105; doi:10.1016/j.jbc.2025.108245)
Supplement: Supporting Information [file mmc1.docx]

Figure S1. Comparison of full-length, wild-type, 0N3R expressing cells to frequently used biosensor cell line (Holmes et al., 2014). HEK293 cells expressing eGFP-0N3R or CFP/YFP fused to the repeat domains of tau with the P301S mutation (Tau RD P301S FRET Biosensor, ATCC CRL-3275) were treated with increasing concentrations of monomeric recombinant tau, 0N4R-P301S^heparin^ tau assemblies, or AD seed. The Tau RD P301S FRET cells respond sensitively but indiscriminately to 0N4R-P301S^heparin^ and AD seed, while the eGFP-0N3R responses specifically to AD seed. Neither cell line show a significant increase above baseline (PBS only) signal with recombinant tau monomer.

Figure S2. Full sequence of HA- (cyan), FLAG- (magenta), or eGFP- (green) tagged 0N3R or 0N4R tau. Linker sequences, grey; microtubule binding region, yellow.
