# Supplementary figures and images for: Sensitive detection and propagation of brain-derived tau assemblies in HEK293-based wild-type tau seeding assays[image]
Source: J Biol Chem. 2025 Jan 27;301(3):108245. doi: 10.1016/j.jbc.2025.108245 (PMC11910105; doi:10.1016/j.jbc.2025.108245)

eGFP-0N3R

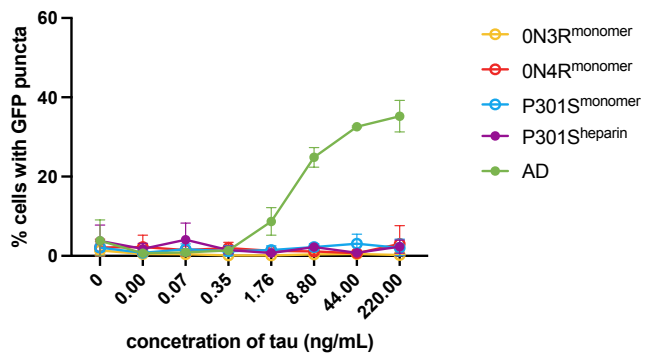

Tau RD P301S FRET Biosensor

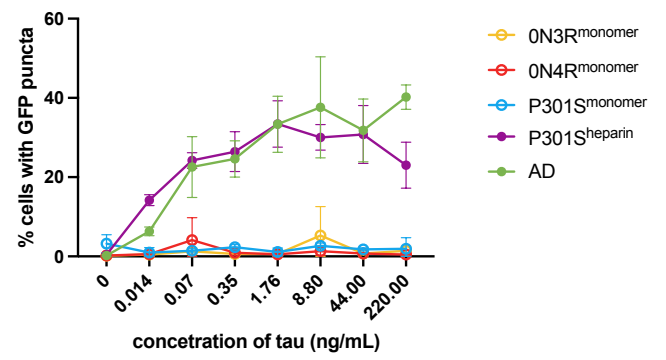

Supplement: Fig S1 [file mmc2.pdf]
